# **Doubling Down:** Will Large Increases in the NIH Budget Promote More Meaningful Medical Innovation?

**DOI:** 10.1017/jme.2023.156

**Published:** 2023

**Authors:** Bhaven N. Sampat

**Keywords:** NIH, Pharmaceuticals, Innovation, Meta-science, Evaluation

## Abstract

Kesselheim proposes doubling the NIH’s budget to promote clinically meaningful pharmaceutical innovation. Since the effects of a previous doubling (from 1998-2003) were mixed, I argue that policymakers should couple future budget growth with investments in experimentation and evaluation.

The paper from Kesselheim makes a strong case for using several policy instruments to promote meaningful medical innovation. One of the instruments he focuses on is public funding. Specifically, he argues for a doubling of the budget of the National Institutes of Health (NIH). The NIH is currently the largest single funder of biomedical research globally. It is composed of 27 Institutes and Centers, with a collective budget of over $45 billion today. A budget doubling would make it a nearly $100 billion agency.

There is some evidence supporting the argument that a doubling would yield more meaningful medical innovation. About a quarter of important drugs have “late-stage” contributions from NIH funding, and for nearly all drugs the data suggest large indirect links to public sector research, including having patents that cite public sector patents and articles related to the drugs. As I have argued elsewhere in this journal^1^, it would be useful to know more about the cruciality of the public sector articles, and the role of complementary private sector contributions, before making confident projections about what increasing NIH funding would do for meaningful drug innovation. Nonetheless, however one looks one can find some links back to NIH research for the majority of important drugs (and presumably other technologies as well, though I don’t think this has been directly studied).

In projecting the likely effects of doubling the NIH’s budget, it also bears recalling this experiment has already been run. Between 1998 and 2003, the agency’s budget increased from about $14 billion to $27 billion ($22 billion to $38 billion in real 2020 dollars).^2^ The doubling was initiated during the Clinton administration and completed under George W. Bush. Powerful legislators across the aisle supported it, as did major scientific and disease advocacy groups and universities. Proponents emphasized a slightly different economic argument than Kesselheim. Not so much that more NIH funding would yield transformative drugs without necessarily incurring the high prices that can result through other major policy instruments (patents, exclusivity, etc), but instead that biomedical research results in drugs and devices that can reduce costly economic losses from disease, and help fuel the growth of life science industry, salient messages given contemporary concerns with health care costs and competitiveness. Advocates circulated poll-tested messages through op-eds across the country, and leading scientific researchers were recruited to help make the case.

Looking back, the effects of the doubling were mixed, at best.^3^ It led to a dramatic expansion of the life science enterprise, growth of academic medical centers, and the hiring of many young researchers often on “soft money” salaries. But these effects were not all positive. With annual budgets, there was pressure to get money out the door during the doubling period. When the party ended in 2003 there was an “extreme hangover”^4^ with a now larger pool of applicants competing for limited grants and jobs, low success rates on NIH applications, and concurrent concerns about hypercompetitiveness in science^5^ and the rigor and integrity of academic medical research itself.^6^

A straightforward solution to this “boom-bust” problem with doubling — which I imagine would be a friendly amendment to Kesselheim’s proposal — would be to move away from “doubling” as a target (at least if what is meant is the rapid doubling in the late 1990s) towards more gradual long-run, sustained increases.

To be sure, part of the problem with the past doubling was that budgets plateaued (or in inflation-adjusted terms, decreased) afterward.^7^ A straightforward solution to this “boom-bust” problem with doubling — which I imagine would be a friendly amendment to Kesselheim’s proposal — would be to move away from “doubling” as a target (at least if what is meant is the rapid doubling in the late 1990s) towards more gradual long-run, sustained increases.^8^

There is a broader issue. The doubling did relatively little to address, and may even have exacerbated, deep-seated structural flaws in the funding system. These include concerns about the conservatism of peer review and its hostility to high-quality ideas, biases against new investigators, administrative burdens facing applicants, and long-standing questions about whether the 75-year-old NIH peer review process is effective at identifying high-quality science or is aligned with the nation’s health priorities.

While counterfactuals are difficult, my instinct is that none of this was good for innovative research or for the development of meaningful therapies. Looking forward, increased funding for medical research may not generate the types of “meaningful” medical innovation Professor Kesselheim and we all seek if mainly focused on business as usual at NIH. There are now growing calls for experimentation around different approaches to the peer review system^9^ and indeed the Center for Medicare and Medicaid Innovation (CMMI) approach to institutionalizing learning and improvement cited in Kesselheim’s testimony may provide a useful model for NIH to try new approaches.^10^

Of course, Kesselheim’s doubling is also accompanied by a call for new ways of doing things. This is a sharp contrast to the previous doubling. In a 1993 *Science* editorial making the case for doubling, prominent scientists (including Harold Varmus, who would go on to become Clinton’s NIH Director and oversee the eventual doubling) argued that NIH-funded research not be concerned with “practical applications” given “the demonstrated ability of the biotechnology and pharmaceutical industries to develop the fruits of basic science.”^11^ Kesselheim’s perspective and argument for a new doubling is different — arguing that at least some of the additional funds go to public funding of late-stage trials, comparative effectiveness studies, and other “practical” studies that help ensure that NIH funded science not only helps generate new drugs, but clinically meaningful drugs that are also affordable. This would represent a significant change in approach and funding strategy. (There are precedents for public sector biomedical research funding in applied biomedical research activities, including in COVIDd-19 vaccine development and during World War II, but these represent the exception not the rule; neither of these specific efforts was primarily via the NIH.)^12^

I and others have also argued for experimentation with more NIH funding for late-stage and applied research.^13^ Economists and science policymakers historically have made the case for public funding of basic research using a “market failure” rationale: the gap between private and social value of the research. Calls for more late-stage public funding recognize that there are likely to be market failures — studies that are socially valuable but not of interest to drug companies — that need addressing in applied research activities as well.

As I have written elsewhere^14^, the NIH budget has increased 1000-fold in real terms since the end of World War II, but knowledge of how to most effectively spend the funds to generate meaningful medical treatments (or for that matter, the best science) has increased very little. So increase the budget, sure, but couple that increase with more investments in learning how to best spend the funds to promote scientific progress, development of meaningful drugs and other innovations, and improved health. This should include experimentation in the peer review process (including, potentially, randomized trials) but also new applied research funding types like those suggested by Kesselheim. It is an opportune time to be considering such changes, given what I think is unprecedented enthusiasm for experimentation in NIH funding models and processes not only among scholars studying the NIH, but also among policymakers, and within the agency itself.

## Note

Dr. Sampat has received grants and contracts from the National Science Foundation, the National Institute for Healthcare Management and the UK Economic and Social Research Council. He has also received payment when writing for the Brookings Institution and the National Bureau of Economic Research.

## References

[1] B.N. Sampat , “The Government and Pharmaceutical Innovation: Looking Back and Looking Ahead.” Journal of Law, Medicine & Ethics 49 no. 1 (2021): 10–18. 10.1017/jme.2021.3 33966646

[2] See *The NIH Almanac: Appropriations (Section 1)*, National Institutes of Health, *available at* <https://www.nih.gov/about-nih/what-we-do/nih-almanac/appropriations-section-1> (last visited November 16, 2023

[3] R.B. Freeman and J. Van Reenen , “Be Careful What You Wish For: A Cautionary Tale About Budget Doubling.” Issues in Science and Technology 25 no. 1 (2008): 27; P. Stephan , How Economics Shapes Science (Cambridge, MA: Harvard University Press, 2012): at 141.

[4] C. Woolston, *Proposed NIH Windfall Raises Hopes — and Fears* (July 27, 2021), Nature, *available at* <https://www.nature.com/articles/d41586-021-02064-x> (last visited November 16, 2023). 10.1038/d41586-021-02064-x 34316037

[5] B. Alberts et al., “Rescuing US Biomedical Research From its Systemic Flaws,” Proceedings of the National Academy of Sciences 111 no. 16 (2014): 5773–5777. 10.1073/pnas.1404402111 PMC4000813 24733905

[6] R. Harris , Rigor Mortis: How Sloppy Science Creates Worthless Cures, Crushes Hope, and Wastes Billions (New York: Basic Books, 2017): at 188–196.

[7] See Freeman and Van Reenan, *supra* note 3.

[8] *Id.*

[9] *Building a Better NIH*, Brookings Institution, *available at* <https://www.brookings.edu/collection/building-a-better-nih/>.

[10] S. Buck and K.T. Kadakia. *Investing In The Science Of Science: What Medicare Can Teach The NIH About Experimentation* (October 21, 2022), Health Affairs Forefront, *available at* < https://www.healthaffairs.org/content/forefront/investing-science-science-medicare-can-teach-nih-experimentation>.

[11] J.M. Bishop, M. Kirschner, and H. Varmus. “Science and the New Administration.” *Science* 259 no. 5094 (1993): 444–445. 10.1126/science.8424162 8424162

[12] D.P. Gross and B.N. Sampat, “Crisis Innovation Policy From World War II to COVID-19.” *Entrepreneurship and Innovation Policy and the Economy* 1 no. 1 (2022): 135–181.

[13] B.N. Sampat, “Whose Drugs are These?.” *Issues in Science and Technology* 36 no. 4 (2020): 42-48; L.L. Ouellette, *Building a Better NIH: Commercialization of NIH-Supported Research* (May 2023), Brookings Institution, *available at* <https://www.brookings.edu/wp-content/uploads/2023/05/LarrimoreOuelletteFinal-3.pdf>.

[14] B. Sampat, *The History and Political Economy of NIH Peer Review* (May 2023), Brookings Institution, *available at* <https://www.brookings.edu/wp-content/uploads/2023/05/SampatFinal-3.pdf>.

